# CircCRIM1 promotes ovarian cancer progression by working as ceRNAs of CRIM1 and targeting miR-383-5p/ZEB2 axis

**DOI:** 10.1186/s12958-021-00857-3

**Published:** 2021-11-30

**Authors:** Yuping Du, Xin Liu, Song Zhang, Shuo Chen, Xue Guan, Qianhui Li, Xi Chen, Yang Zhao

**Affiliations:** 1 Department of Obstetrics and Gynecology, Department of Gynecologic Oncology Research Office, Key Laboratory for Major Obstetric Diseases of Guangdong Province, The Third Affiliated Hospital of Guangzhou Medical University, Guangzhou, 510150 China; 2 Department of Thoracic Surgery, The First Affiliated Hospital of China Medical University, Department of Environmental and Occupational Health, School of Public Health, China Medical University, Shenyang, 110001 China; 3 Department of Gynecology, The First Affiliated Hospital of China Medical University, Shenyang, 110001 China

**Keywords:** circCRIM1, CRIM1, Ovarian cancer, miR-145-5p, miR-383-5p

## Abstract

**Background:**

Ovarian cancer is the leading cause of death in patients with gynecologic cancer, and circular RNAs (circRNAs) are involved in cancer progression. However, there are limited studies on the roles of circRNAs in ovarian cancer.

**Methods:**

We designed divergent and convergent primers, used sanger sequencing and RNase R digestion to verify the source of circCRIM1. We detected the expression of circCRIM1 and its parental gene cysteine rich transmembrane BMP regulator 1 (CRIM1) in ovarian cancer and normal ovarian samples via qRT-PCR. MTT viability assay, apoptosis assay, wound healing assay and invasion assay were used to investigate the function of circCRIM1 and CRIM1 in ovarian cancer cell lines OVCAR3 and CAOV3. Mice xenografts experiment was performed. Bioinformatics predicted the microRNAs that bond with circCRIM1 and CRIM1, and dual luciferase reporter system confirmed it. Rescue experiments of microRNAs mimics transfection on the basis of circCRIM1 over-expression were carried out to uncover the mechanism by which circCRIM1 played cancer-promoting roles in ovarian cancer.

**Results:**

CircCRIM1 was derived from CRIM1 by back-splicing. CircCRIM1 and CRIM1 had higher expression in ovarian cancer than in normal ovarian tissues, and both of them promoted ovarian cancer progression in vitro. In vivo circCRIM1 promoted the growth of tumors. CircCRIM1 and CRIM1 had a positive correlation relationship in the same cohort of ovarian cancer tissues. Bioinformatics predicted and dual luciferase assay confirmed circCRIM1 and CRIM1 bond with miR-145-5p, and circCRIM1 bond with miR-383-5p additionally. CircCRIM1 positively affected the expression of CRIM1. After circCRIM1 was over-expressed, miR-145-5p mimics transfection reversed the expression of CRIM1. Western blot discovered circCRIM1 positively affected the expression of zinc finger E-box binding homeobox 2 (ZEB2). Rescue experiments found miR-383-5p mimics reversed ZEB2 expression and the cancer-promoting effects of circCRIM1.

**Conclusions:**

CircCRIM1 bond with miR-145-5p to work as competing endogenous RNA (ceRNA) of CRIM1, and circCRIM1 bond with miR-383-5p to improve the expression of ZEB2 in ovarian cancer. CircCRIM1 and CRIM1 promoted the ovarian cancer progression and supplied a novel insight into the researches of ovarian cancer.

**Supplementary Information:**

The online version contains supplementary material available at 10.1186/s12958-021-00857-3.

## Background

Ovarian cancer is the leading cause of death in patients with gynecologic cancer. Owing to the lack of an early diagnostic strategy, this seriously declines survival. Especially in serous ovarian cancer, majority of patients are diagnosed at stage III (51%) or IV (29%) [1–3]. Therefore, it is extremely important to seek early markers of ovarian cancer.

Circular RNAs (CircRNAs) working as a novel class of non-coding RNAs, played critical roles during the process of initiation and development of cancers. They consisted of a circular configuration through typical 5′ to 3′ - phosphodiester bond. Lacking free terminus, thus they gained much more stable existence in the cells compared to linear RNAs [4]. To date, the researches on the roles of circRNAs have been continuously extended and deepened, which was involved in multiple diseases, including cancers [5, 6], cardiovascular diseases [7, 8], diabetes [9], autoimmune diseases and so on [10]. CircRNAs exerted their function through multiple ways. They could regulate the expression of their parental genes by transcriptional and post-transcriptional pathways, act as microRNAs “sponges”, bind with RNA-binding proteins to form RNA-protein complexes and even have open reading frames (ORFs) to be translated into proteins/peptides [11–16]. According to the previous researches, many circRNAs have identified their important roles in the cancers [17–19].

CircRNAs were mainly derived from precursor mRNAs by the way of back-splicing [20–23]. Hsa_circ_0002346 (referred to as circCRIM1) was derived from exon2–4 of cysteine rich transmembrane BMP regulator 1 (CRIM1) that was a bone morphogenetic protein family antagonist [24, 25]. In the existing studies, CRIM1 had a higher expression in gastric cancer and drug-resistant myeloid leukemia cells [26, 27]. Furthemore, CRIM1 promoted adhesion and migration of lung cancer cells and promoted invasion of prostate cancer cells [28, 29]. However, there was no comparative report about the function of CRIM1 and circCRIM1 in ovarian cancer. Therefore, this paper focused on the role and mechanism of circCRIM1 and its parental gene coding protein CRIM1 in ovarian cancer.

## Methods

### Specimens collection

Normal ovarian (*n* = 24) and ovarian cancer tissues (*n* = 130) were collected from surgical excision in the First Affiliated Hospital of China Medical University (Shenyang, Liaoning, China), and patients had no previous radiotherapy or chemotherapy. This project was approved by the ethic committee of China Medical University (No:2018–132) and written consents were obtained from all patients or their families.

### Cell lines and transfection

The human ovarian cancer cell lines OVCAR3 and CAOV3 were cultured in RPMI 1640 (HyClone, Logan, UT, USA) added with 10% FBS and 1% penicillin/streptomycin. All siRNAs, plasmids and microRNA mimics transfection used Lipofectamine 3000 reagent (Invitrogen, Carlsbad, USA). The sequences of siRNA of CRIM1 (Sigma-Aldrich, St. Louis, MO, USA): sense CUCAGUACUCCCUCCAUUUdTdT, anti-sense AAAUGGAGGGAGUACUGAGdTdT. The detail of plasmids was shown in the Tables S5, S6, S7, S8.

### RNase R

Total RNA was extracted from OVCAR3, then 5 μg of total RNA was formulated into a 20 μl system with RNase R (Epicentre, Madison, USA) for RNase R (+) group and RNase-Free water for RNase R (−) group, and incubated at 37 °C for 0, 10, 20, 30 min. The reaction was terminated at 70 °C for 10 min. The 5 μl of each product was reverse transcribed and amplified following qRT-PCR procedures.

### qRT-PCR

Total RNA was extracted from tissues or cells using RNA isolater Total RNA Extraction Reagent (Vazyme Biotech, Nanjing, China) and then was reverse transcribed to cDNA with reverse transcription kit (Promega, Madison, Wisconsin, USA). cDNA was amplified using 2xSYBR Green qPCR Master Mix (Bimake, Houston, TX, USA). 18 s or β-actin was utilized to normalize the expression. The detail of primer sequences was shown in the Tables S9.

### MTT viability assay

Cells were seeded in 96-well plates at a density of 3000 cells/well. Cells were transfected after overnight, if it needed. And then each well added MTT (5 mg/ml) 20 μl at indicated time periods. After further incubation of 4 h, culture medium was removed and 150 μl DMSO was added. The absorbance was obtained at 490 nm using a microplate spectrophotometer (BioTek Instruments, Winooski, VT).

### Apoptosis assay

Cells were collected and washed with PBS. 100 μl of 1 × buffer, 5 μl of Annexin V-FITC and 5 μl of PI (BD Biosciences, San Jose, CA, USA) were used in the dark for transient transfected cells. And for stable transfected cells, 7AAD and annexin V-PE (BD Biosciences) were used.

### Wound healing assay

Cells were seeded in 6-well plate. And after overnight scratched it with a 200 μl tip. Cells were cultured in 5% serum medium after washed away floating cells with PBS. Cells were transfected if it needed. Cells migration was observed and photographed at 0 h and 48 h. The wound healing rate was measured by the way as follows: (area at 0 h − area of wound at 48 h)/ area at 0 h.

### Invasion assay

Cells diluted by serum-free medium were seeded in the top compartment of the Transwell cell culture chambers (BD Bioscience) at a density of 4 × 10^4^ cells per well, and precoated membrane with matrigel (1:10) before inoculation. Complete medium 600 μl was layered in the bottom compartment. Cells were transfected if it needed. After being incubated 48 h, the cells were fixed and dyeing.

### Xenografts assay

All 4-week-old female BALB/c nude mice (Vital River Laboratories, Beijing, China) were randomly divided into two groups (*n* = 6) and were fed under the standard condition. The vector and plasmid transfected cells were injected subcutaneously at a concentration of 1 × 10^7^ cells/ 200 μl. The tumor volumes were monitored and mice were sacrificed when the maximum tumor diameter was close to 2 cm. Tumor volumes were calculated using the formula: (length × width^2^) / 2. All animal experiments conformed to the National Institutes of Health Guide for the Care and Use of Laboratory Animals and were supported by the China Medical University Animal Care and Use Committee.

### Western blotting

Cells were lysed by RIPA buffer and the concentration was determined. Proteins were run on sodium dodecyl sulphate-polyacrylamide gels relying on molecular weight and then were wet transferred to Hybond membranes. 5% fat-free milk was used to block nonspecific combination at room temperature for 2 h, followed by incubation at 4 °C overnight with primary antibodies against CRIM1(Cat No. bs-21654R, Biosis, China), Flag (Cat No. F3165, Sigma-Aldrich), ZEB2(Cat No. 14026–1-AP, Proteintech, China) and β-actin (Cat No. 20536–1-AP, Proteintech). TBST washed membranes 4 times and were incubated with the secondary antibody for 2 h at room temperature. An enhanced chemiluminescence (Santa Cruz Biotechnology, Santa Cruz, CA, USA) was used to visualize the bands.

### Dual luciferase assay

The sequences of wild type circCRIM1 and microRNA binding sites mutant circCRIM1 were inserted into dual luciferase reporter plasmid psicheck2.0 (HANBIO, Shanghai, China). Similarly, the wild type sequences of CRIM1 mRNA 3’UTR and mutant sequences were inserted into vector GV272 (GENE, Shanghai, China). MicroRNA mimics or miR-NC was co-transfected with above wild and mutant plasmids into HEK293 cells. After 48 h, Dual-Luciferase Reporter System (Promega) was used to measure luciferase activities.

### Statistical analyses

All data were expressed as mean ± standard deviation. At least three independent experiments were performed. Two groups were compared with the two-sided student’s t test. Correlation was calculated using Pearson’s correlation analysis.

## Results

### CircCRIM1 is derived from CRIM1 by back-splicing

CircCRIM1 with a 538 nt circular structure was predicted by CircRNA Db to be derived from CRIM1 exon2–4 (Fig. S1a). The back-splicing junction was amplified with divergent primers and verified by Sanger sequencing (Fig. S1b). CircCRIM1 was more resistant to RNase R than CRIM1 and 18 s (Fig. S1c; *p* < 0.05), thus confirmed its ring structure. We designed 2 pairs of convergent primers and one pair of divergent primers to amplify CRIM1 and circCRIM1 from cDNA and gDNA and verified circCRIM1 was only derived from RNA but not DNA (Fig. S1d). These results suggested that circCRIM1 was derived from CRIM1 by back-splicing.

### Both circCRIM1 and CRIM1 are up-regulated in ovarian cancer

**Fig. 1:**
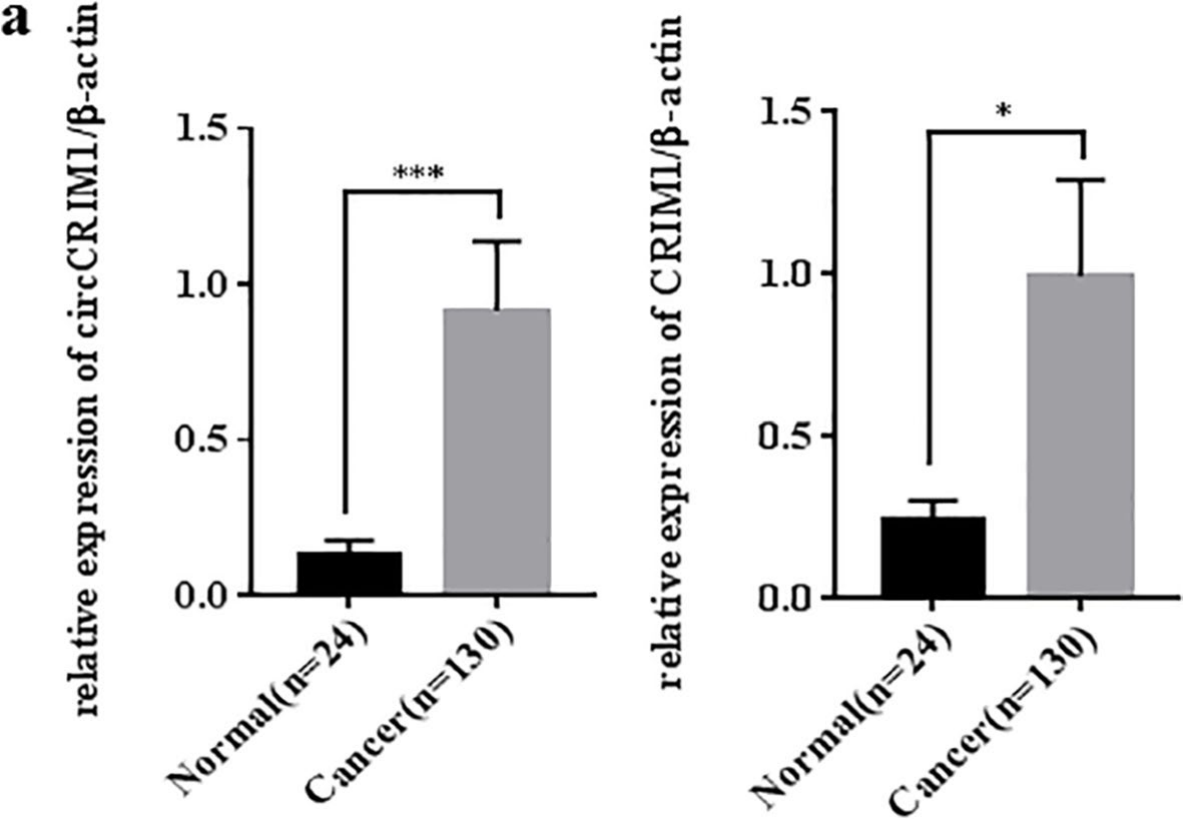
CircCRIM1 and CRIM1 are up-regulated in ovarian cancer. Both circCRIM1 and CRIM1 were up-regulated in ovarian cancer (**a**). Data are shown as the mean ± SD. *P < 0.05, **P < 0.01, ***P < 0.001 and ****P < 0.0001

The expression of circCRIM1 was significantly up-regulated in ovarian cancer tissues compared with normal ovarian tissues. Furthemore, we detected CRIM1 expression in the same cohort of ovarian cancer tissues and normal ovarian tissues, the expression of CRIM1 mRNA was higher in ovarian cancer tissues than that in normal ovarian tissues (Fig. 1a; *p* < 0.05). The detail of expression information was shown in the Tables S1, S2, S3, S4.

### CircCRIM1 promotes the progression of ovarian cancer in vitro

**Fig. 2:**
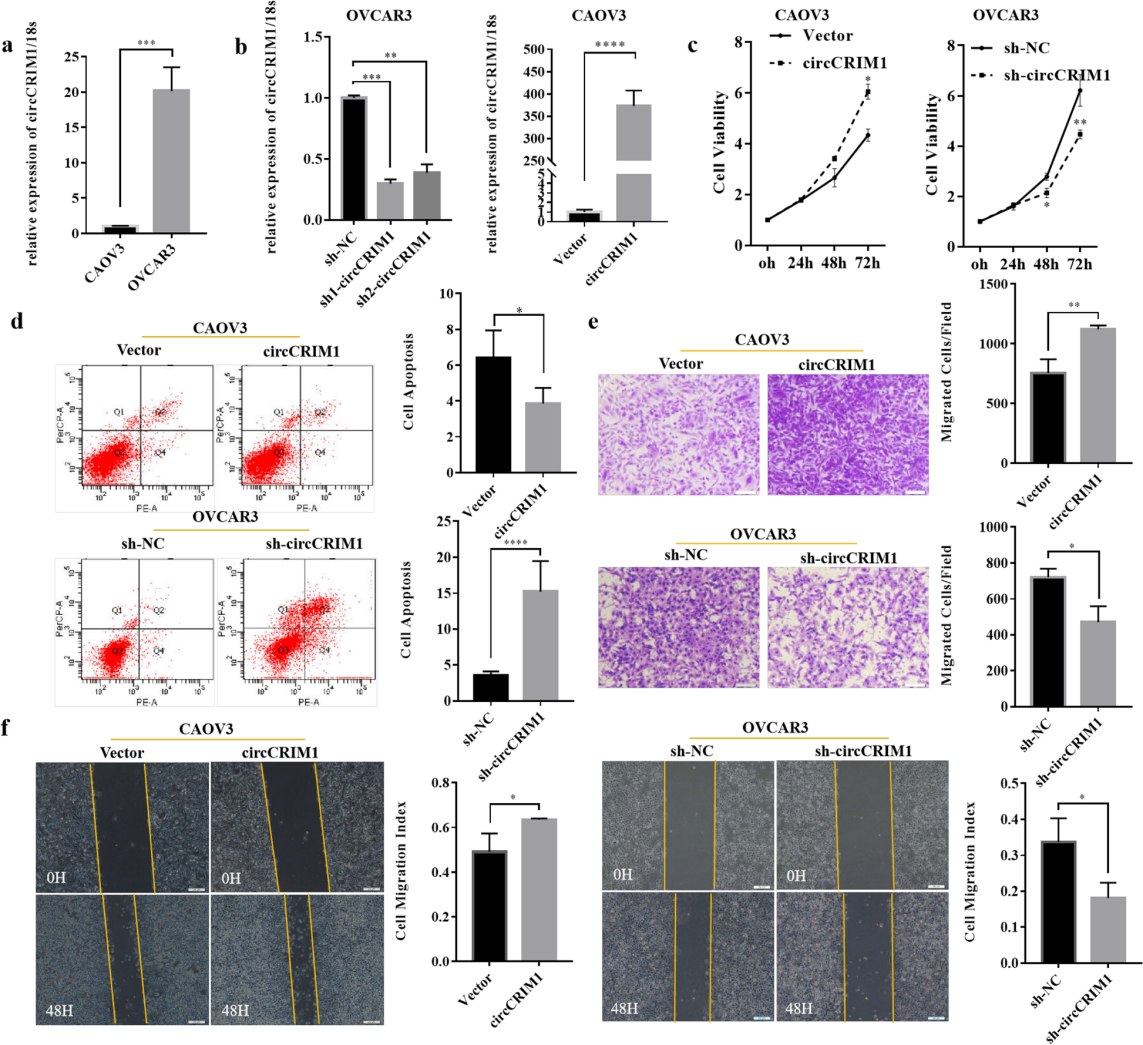
CircCRIM1 promotes the progression of ovarian cancer in vitro. Ovarian cancer cell line OVCAR3 had a higher expression of circCRIM1 than CAOV3 (**a**). The over-expression and knockdown plasmids of circCRIM1 were examined by qRT-PCR (**b**). Enhanced expression of circCRIM1 promoted cell viability, migration, invasion and inhibited apoptosis. CircCRIM1 knockdown had opposite effect (**c**, **d**, **e** and **f**). Three separate experiments were conducted; data are shown as the mean ± SD. *P < 0.05, **P < 0.01, ***P < 0.001 and ****P < 0.0001

To evaluate the functional role of circCRIM1 in ovarian cancer, circCRIM1 over-expression and knockdown plasmids were respectively constructed. Ovarian cancer cell line OVCAR3 had a higher expression of circCRIM1 than ovarian cancer cell line CAOV3 (Fig. 2a; *P* < 0.05). The knockdown plasmid sh1 had stronger suppression effect and we used it for the following experiments. The over-expression plasmid was transfected into CAOV3 and verified with qRT-PCR (Fig. 2b; *P* < 0.05). Enhanced expression of circCRIM1 promoted cell viability, migration, invasion and inhibited apoptosis. On the other hand, circCRIM1 knockdown inhibited cell viability, migration, invasion and promoted apoptosis (Fig. 2c, d, e and f; *P* < 0.05).

### CircCRIM1 promotes the progression of ovarian cancer in vivo

**Fig. 3:**
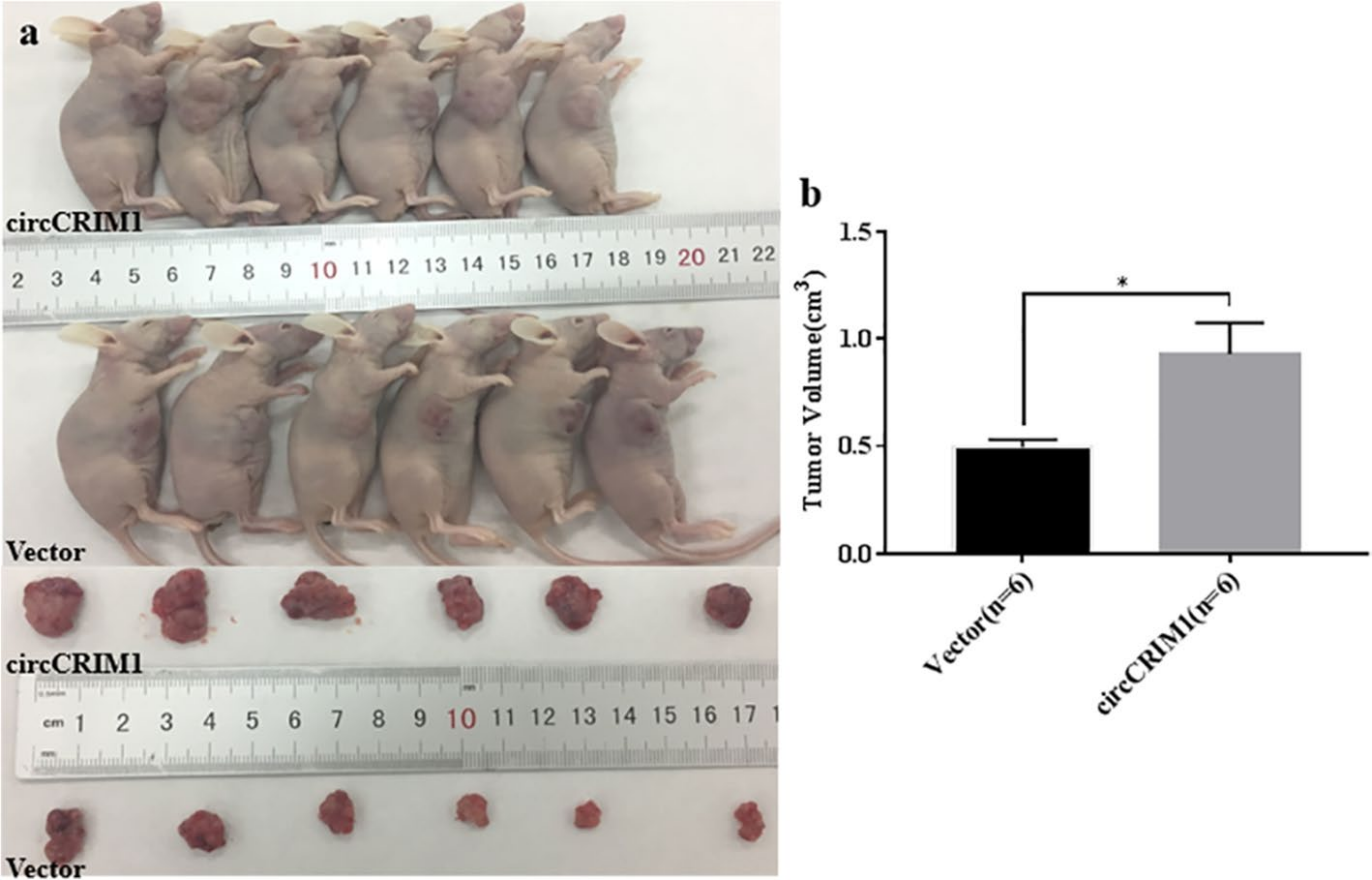
CircCRIM1 promotes the growth of ovarian cancer in vivo. The group that upregulated circCRIM1 had more bulky tumor volume compared to vector group (**a** and **b**). Data are shown as the mean ± SD. *P < 0.05, **P < 0.01, ***P < 0.001 and ****P < 0.0001

In order to investigate the role of circCRIM1 in tumor growth in vivo, the CAOV3 cells transfected with vector or circCRIM1 over-expression plasmid were respectively subcutaneously injected into the right flanks of mice. The tumor volumes of circCRIM1 over-expression group were larger than that of control group (Fig. 3a and b; *P* < 0.05). The result revealed that circCRIM1 promoted the tumor growth in vivo.

### CRIM1 promotes the progression of ovarian cancer

**Fig. 4:**
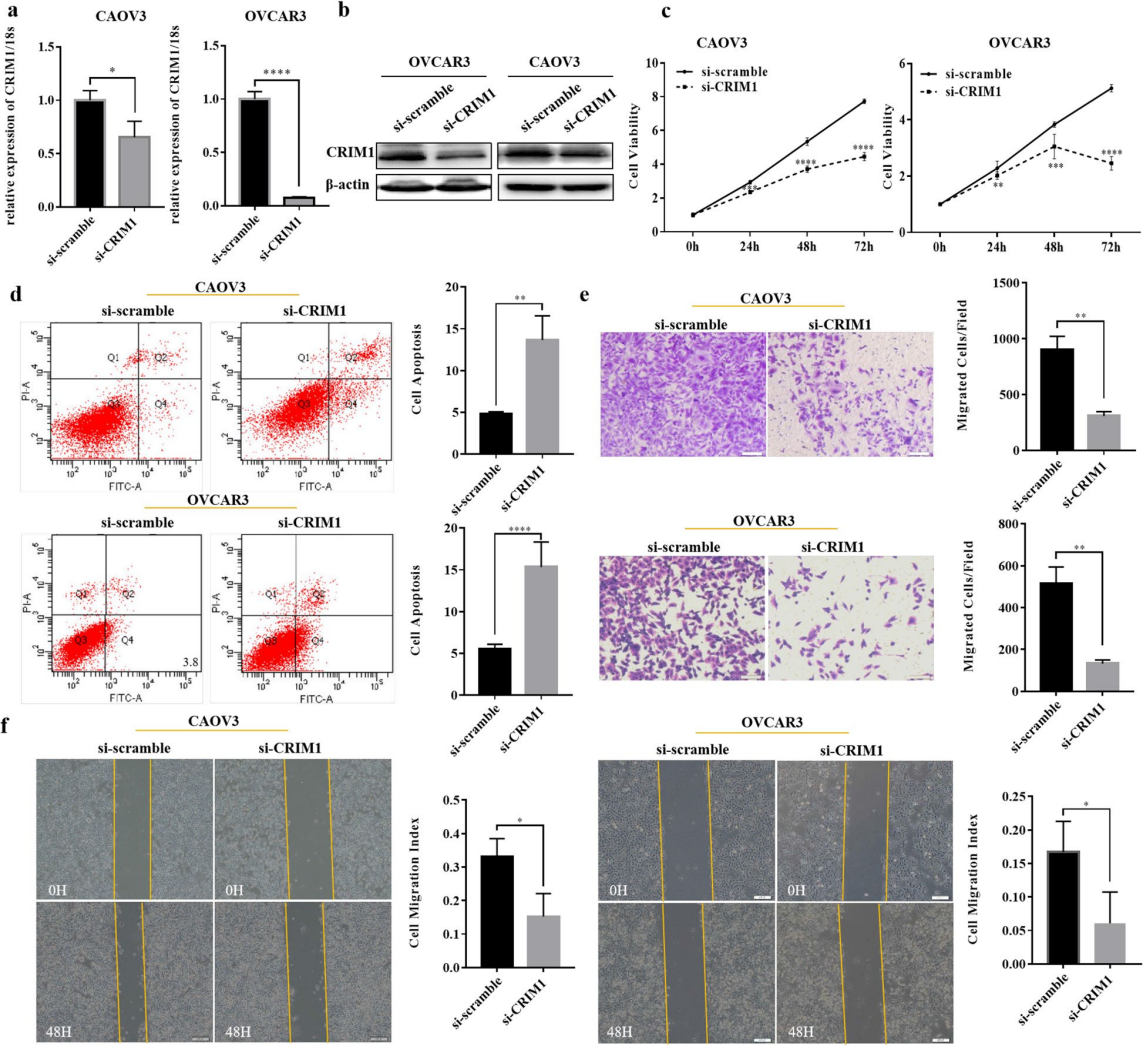
CRIM1 promotes ovarian cancer progression. The siRNA of CRIM1 was examined by qRT-PCR and western blot (**a** and **b**). CRIM1 knockdown decreased the viability, migration, invasion ability of ovarian cancer cell lines and increased apoptic rate (**c**, **d**, **e** and **f**). Three separate experiments were conducted; data are shown as the mean ± SD. *P < 0.05, **P < 0.01, ***P < 0.001 and ****P < 0.0001

Through design of siRNA, CRIM1 was knocked down in ovarian cancer cell lines CAOV3 and OVCAR3. Interference efficiency of CRIM1 siRNA was verified with qRT-PCR and western blot (Fig. 4a and b; *P* < 0.05). After CRIM1 was interfered, the viability, migration, invasion ability of ovarian cancer cell lines decreased and apoptic rate increased (Fig. 4c, d, e and f; *P* < 0.05).

### CircCRIM1 positively regulates the expression of CRIM1 through miR-145-5p

**Fig. 5:**
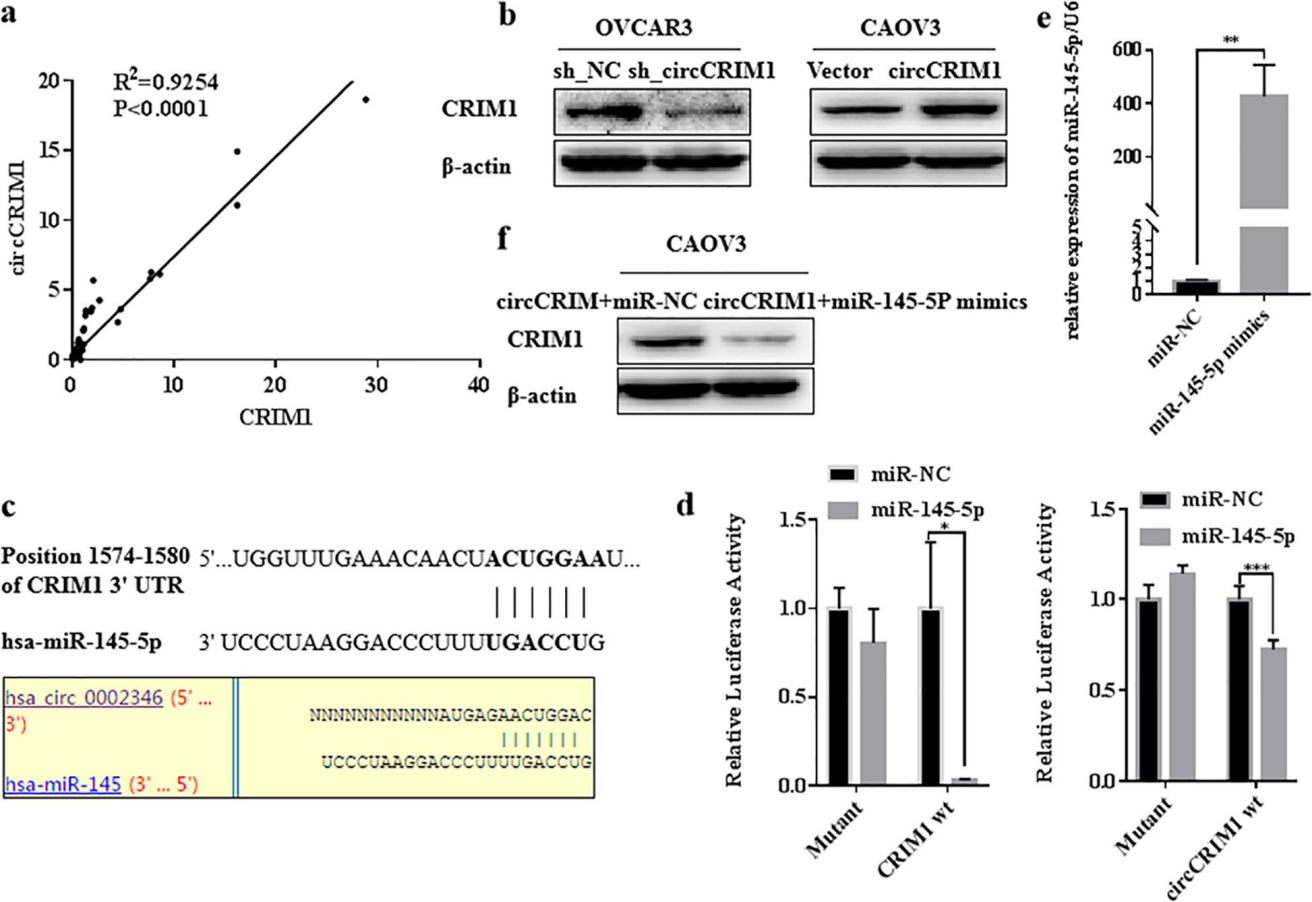
CircCRIM1 positively regulates the expression of CRIM1 through miR-145-5p. QRT-PCR showed that there was a positive correlation relationship between circCRIM1 and CRIM1 (**a**). CRIM1 expression was obviously positively influenced by circCRIM1 (**b**). TargetScanHuman predicted miR-145-5p might combine with 3’UTRs of CRIM1 and Circular RNA Interactome predicted miR-145-5p might bind with circCRIM1(**c**). Dual luciferase reporter assay verified it (**d**). MiR-145-5p treatment reversed the expression of CRIM1 after the over-expression of circCRM1(**f**). MiR-145-5p mimics efficiency was verified (**e**). Three separate experiments were conducted; data are shown as the mean ± SD. *P < 0.05, **P < 0.01, ***P < 0.001 and ****P < 0.0001

We analyzed the expression of CRIM1 mRNA and circCRIM1 in the same cohort of ovarian cancer tissues, and found that there was a positive correlation relationship between circCRIM1 and CRIM1 (Fig. 5a; *P* < 0.05). We also found that CRIM1 expression was positively influenced by circCRIM1 (Fig. 5b). TargetScanHuman predicted that miR-145-5p was likely to combine with 3’UTR of CRIM1 and Circular RNA Interactome predicted miR-145-5p had complementary sequences targeting circCRIM1 (Fig. 5c). Dual luciferase reporter assay showed that the co-transfection of miR-145-5p mimics and 3’UTR fragment of CRIM1 inhibited luciferase activity compared to miR-NC and 3’UTR of CRIM1 co-transfection, while miR-145-5p binding site mutation abolished this effect. Similarly, miR-145-5p mimics and wild type circCRIM1 reporter plasmid co-transfection inhibited the luciferase activity compared to miR-NC and wild type circCRIM1 co-transfection, while miR-145-5p binding site mutation abolished this effect (Fig. 5d; *P* < 0.05). Furthermore, miR-145-5p mimics treatment reversed the expression of CRIM1 after over-expression of circCRM1, and miR-145-5p mimics over-expression ability was verified (Fig. 5e and f; *p* < 0.05). These results indicated that the expression of CRIM1 was positively regulated by circCRIM1 through miR-145-5p.

### CircCRIM1 may encode 188aa protein

**Fig. 6:**
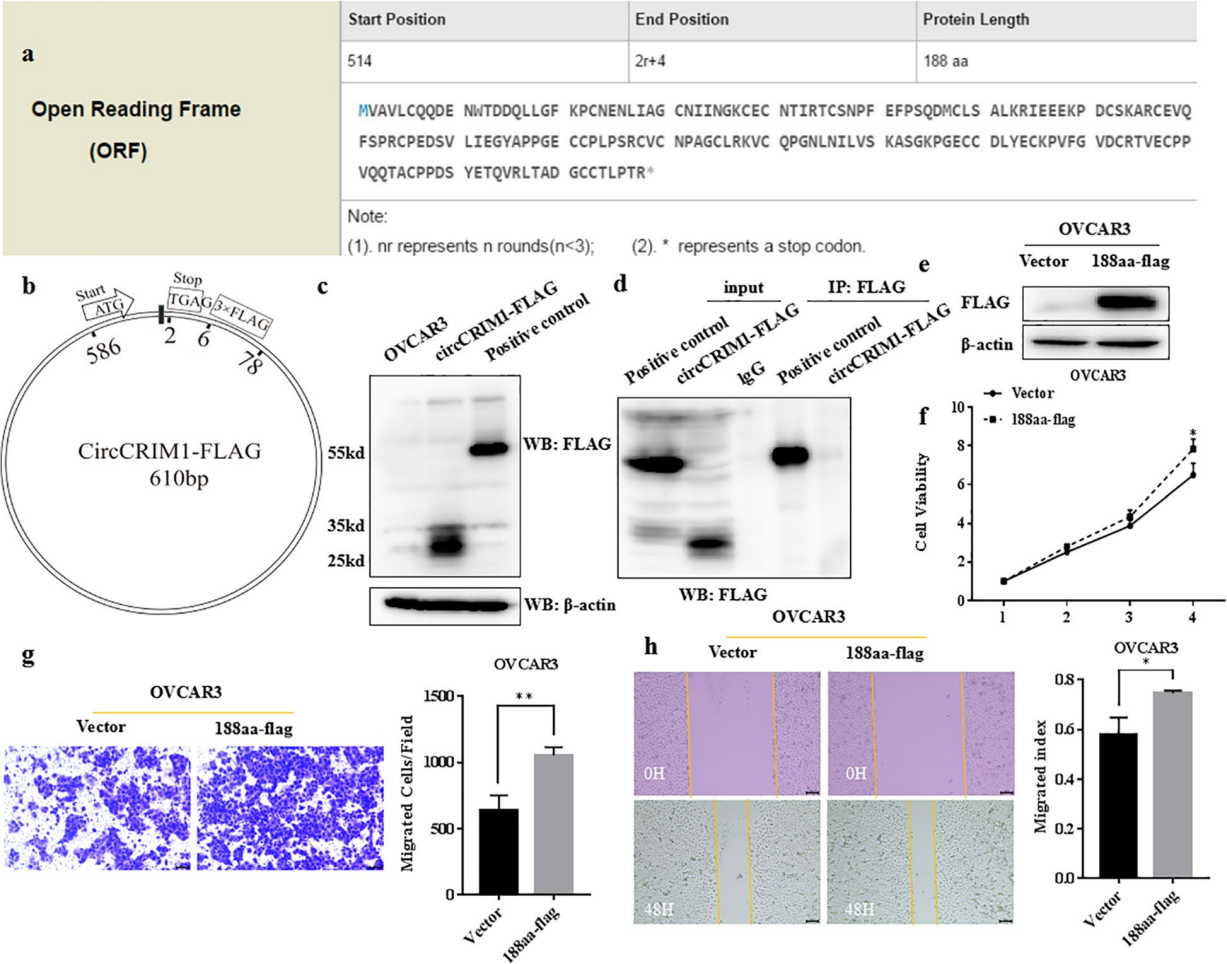
CircCRIM1 may encode 188aa protein. CircRNA Db predicted circCRIM1 had ORF with size 188aa (**a**). 3 × flag sequences were added into putative ORF in original circCRIM1 over-expression plasmid (**b**). Flag antibody was used to detect the expression of coding protein-3 × flag fusion protein, and there was detectable protein band (**c**). When the positive control was pulled down, 188aa-3 × flag failed to be pulled down by means of immunoprecipitation with flag antibody (**d**). CRIM1-188aa and 3 × flag tag were inserted into the protein expression vector and transfected into OVCAR3. Western blot verified the expression (**e**). CRIM1-188aa over-expression promoted the viability, migration and invasion of OVCAR3 (**f**, **g** and **h**). Three separate experiments were conducted; data are shown as the mean ± SD. *P < 0.05, **P < 0.01, ***P < 0.001 and ****P < 0.0001

CircRNA Db predicted that circCRIM1 had a putative ORF with size 188aa (Fig. 6a). To explore that whether circCRIM1 could encode protein, 3 × flag sequences were added into putative ORF in original circCRIM1 over-expression plasmid and avoided frame shifting, stop codon disruption, junction disruption and IRES disruption (Fig. 6b). We used flag antibody to detect the expression of 188aa-3 × flag fusion protein with predicted molecular weight around 27kd. There was detectable protein band (Fig. 6c). We were trying to pull down 188aa-3 × flag for mass spectrometry verification by means of immunoprecipitation with flag antibody. However, when the positive control was pulled down, 188aa-3 × flag was unable to be pulled down (Fig. 6d). In spite of this, 188aa and 3 × flag sequences were introduced into protein expression vector and transfected into OVCAR3 (Fig. 6e). We found over-expression of 188aa-flag promoted cell proliferation, invasion and migration in OVCAR3 (Fig. 6f, g and h; *P* < 0.05).

### CircCRIM1 binds with miR-383-5p to promote ovarian cancer progression

**Fig. 7:**
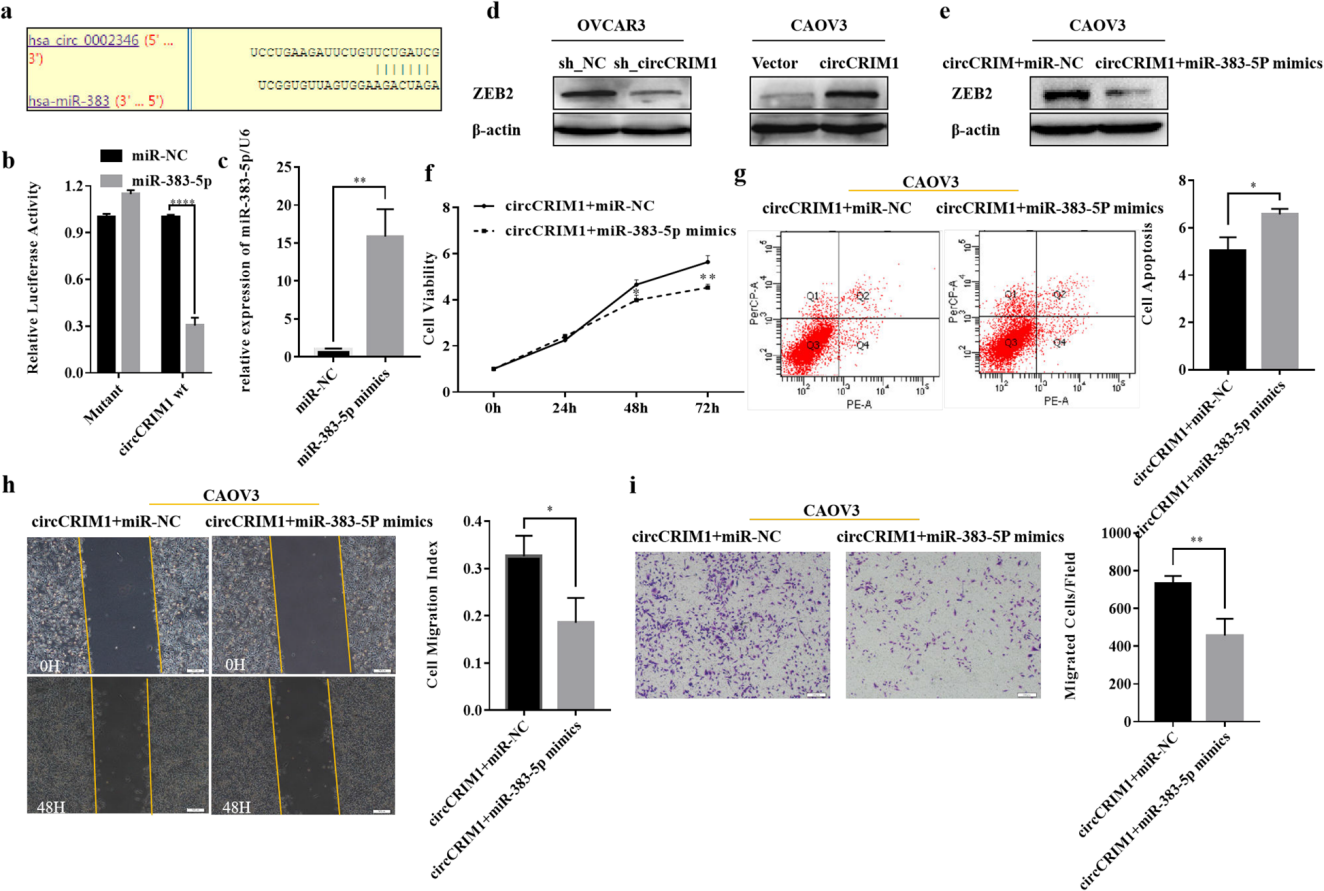
CircCRIM1 binds with miR-383-5p to promote ovarian cancer progression. Circular RNA Interactome predicted miR-383-5p might bind with circCRIM1 and the dual luciferase reporter assay was performed to verify it (**a** and **b**). MicroRNA mimics over-expression ability was verified (**c**). CircCRIM1 enhancement or knockdown has positively effect on ZEB2 (**d**). MiR-383-5p mimics treatment after circCRIM1 over-expression reversed the promotion effect of circCRIM1 on viability, migration, invasion ability and ZEB2 expression, similarly increased apoptic rate (**e**, **f**, **g**, **h** and **i**). Three separate experiments were conducted; data are shown as the mean ± SD. *P < 0.05, **P < 0.01, ***P < 0.001 and ****P < 0.0001

By using Circular RNA Interactome, we identified that miR-383-5p had complementary sequences targeting to circCRIM1 (Fig. 7a). The dual luciferase reporter assay was performed and found that miR-383-5p mimics and wild type circCRIM1 reporter plasmid co-transfection inhibited luciferase activity compared to miR-NC and wild type circCRIM1 co-transfection, while miR-383-5p binding site mutation abolished this effect (Fig. 7b; *P* < 0.05). MiR-383-5p mimics over-expression ability was verified (Fig. 7c; *P* < 0.05). MiR-383-5p has been reported to bind with ZEB2. Western blot found circCRIM1 enhancement or knockdown positively regulated ZEB2 (Fig. 7d). MiR-383-5p mimics treatment in CAOV3 that over-expressed circCRIM1 reversed the promotion effect of circCRIM1 on viability, migration, invasion ability and ZEB2 expression, increased apoptic rate (Fig. 7e, f, g, h and i; *P* < 0.05).

## Discussion

By the way of cDNA and gDNA PCR, Sanger sequencing and RNase R digestion, we confirmed circCRIM1 was derived from exon2–4 of protein coding gene CRIM1 pre-mRNA by back-splicing. In our study, we identified that circCRIM1 was up-regulated in ovarian cancer compared with normal ovarian tissues. In order to determine the function of circCRIM1 in ovarian cancer, we detected the expression levels of circCRIM1 in ovarian cancer cell lines CAOV3 and OVCAR3. We found that the circCRIM1 was higher in OVCAR3 than that in CAOV3. Thus, we chose CAOV3 to over-express and OVCAR3 to knock down the expression of circCRIM1. Functional experiments were carried out to explore the roles of circCRIM1 in ovarian cancer. We observed that circCRIM1 enhancement promoted cell viability, migration, invasion and inhibited apoptosis and circCRIM1 knockdown produced opposite effects. The nude mice xenograft assay was performed and suggested that circCRIM1 promoted the growth of tumors in vivo. All in all, circCRIM1 promoted the progression of ovarian cancer in vitro and in vivo.

A few articles have revealed the cancer-promoting effects of CRIM1, but there was no comparative study on the effects of CRIM1 in ovarian cancer. We detected the CRIM1 mRNA in the same samples of ovarian cancer and normal ovarian tissues as above. The results revealed that CRIM1 mRNA was higher in ovarian cancer tissues compared to normal ovarian tissues. Then we identified that CRIM1 interference inhibited cell viability, migration, invasion and promoted apoptosis in ovarian cancer cell lines. Thus, CRIM1 and circCRIM1 both played cancer-promoting roles in ovarian cancer.

As mentioned above, circRNAs could regulate the expression of their parental genes. We analyzed the expression levels of circCRIM1 and CRIM1 in ovarian cancer samples and found there was a positive correlated relationship between circCRIM1 and CRIM1 mRNA. Apart from this, the over-expression and knockdown of circCRIM1 in ovarian cancer cell lines positively influenced CRIM1 expression. So how did circCRIM1 regulate the expression of CRIM1? We intersected microRNAs predicted by TargetScanHuman to bind to CRIM1 mRNA 3’UTR and microRNAs predicted by Circular RNA Interactome to bind to circCRIM1. It was found that miR-145-5p might bind to both CRIM1 mRNA 3’UTR and circCRIM1 [30–33]. Previous studies have found that the expression of miR-145-5p in normal ovarian tissues is greater than in epithelial ovarian cancer, and up-regulated expression of miR-145-5p significantly inhibited proliferation, invasion and in vivo tumor formation, and induced apoptosis in ovarian cancer cell lines [34, 35]. And then the dual luciferase assay was performed to verify the combination of miR-145-5p and CRIM1 mRNA 3’UTR or circCRIM1. Furthermore, miR-145-5p mimics treatment decreased the expression of CRIM1 in the cells that over-expressed circCRIM1. Thus, it indicated that circCRIM1 worked as ceRNA of CRIM1 mRNA trough miR-145-5p.

Recent studies have proposed that some circRNAs containing ORFs could be efficiently translated into proteins or peptides. Circ-AKT3 encoded a new 174aa protein, which negatively regulated the PI3K / AKT pathway [36]. Circ-FBXW7 encoded a 185aa protein that reduced the half-life of c-Myc [37]. We sought to elucidate that whether circCRIM1 had the ability of encoding protein or peptide. CircRNA Db predicted that circCRIM1 with ORF started from 514 nt had the possibility to produce a protein with length 188aa [38]. We added 3 × flag sequences into putative ORF in original circCRIM1 over-expression plasmid and used flag antibody to hybrid with predicted 188aa-flag fusion protein which had a molecular weight about 27kd. There was detectable protein band. Then we tried to pull down 188aa-3 × flag for mass spectrometry verification by means of immunoprecipitation with flag antibody to confirm the existence of 188aa. However, when the positive control was pulled down, 188aa-3 × flag failed to be pulled down. Its not clear if this was due to factors that the flag sequence was included in the 188aa sequence, the stability of the protein was poor, or something else. It required further exploration. In spite of this, 188aa and 3 × flag tag were inserted into the protein expression vector and transfected into OVCAR3. The over-expression of 188aa promoted cell viability, migration and invasion.

CircRNA usually exerted its function through the way of “sponge”. Bioinformatic analyses indicated that circCRIM1 might target with miR-383-5p. By means of the dual luciferase assay, we confirmed that miR-383-5p combined with circCRIM1. Previous study found that miR-383-5p worked as tumor suppressor gene to suppress proliferation of ovarian cancer cells and inhibit progression of gastric cancer [39–41]. While ZEB2 worked as oncogenic genes in ovarian cancer to drive EMT transition, tumorigenesis and peritoneal metastasis [42, 43]. The study found that ZEB2 expression in ascites of patients with high-grade serous ovarian cancer was higher than that of primary ovarian cancers. ZEB2 positively regulated the proportion of CD133+ cancer stem-like cells in epithelial ovarian cancer cells and promote the tumorigenesis and peritoneal metastasis capacity of CD133+ cancer stem-like cells. Bioinformatics, luciferase reporter system and western blot verified ZEB2 mRNA 3’UTR was the target of miR-383-5p [44]. Western blot was performed and we found the expression of ZEB2 was consistent with circCRIM1. Then rescue experiments were carried out. We found that miR-383-5p mimics transfection after circCRIM1 over-expression reversed the promotion of circCRIM1 on cell viability, migration, invasion and ZEB2 expression, and promoted apoptosis. Taken together, circCRIM1 bond with miR-383-5p to up-regulate ZEB2 in ovarian cancer and played the cancer-promoting roles. In addition, in the following work, we could focus on the effect of circCRIM1 on ovarian cancer peritoneal metastasis.

In a word, circCRIM1 worked as ceRNA of CRIM1 through miR-145-5p, and bond with miR-383-5p to improve ZEB2 to play the cancer-promoting roles in ovarian cancer.

## Conclusion

This article supplied a significant perspective on the roles of circCRIM1 in ovarian cancer. It bond with miR-383-5p to improve the expression of ZEB2 in ovarian cancer. Moreover, CRIM1, the parental gene of circCRIM1, promoted the progression of ovarian cancer. CircCRIM1 bond with miR-145-5p to work as ceRNA of CRIM1, and both of them offered a novel insight into researches of ovarian cancer.

## Supplementary Information

**Additional file 1 Supplemental Table 1.** circCRIM1 expression in normal ovarian and ovarian cancer tissues. The detail information of circCRIM1 expression in normal ovarian and ovarian cancer tissues. **Supplemental Table 2.** CRIM1 expression in normal ovarian and ovarian cancer tissues. The detail information of CRIM1 expression in normal ovarian and ovarian cancer tissues. **Supplemental Table 3.** Correlation of circCRIM1 expression with different clinicopathological features of ovarian cancer. The detail information of the correlation of circCRIM1 expression with different clinicopathological features of ovarian cancer. **Supplemental Table 4.** Correlation of CRIM1 expression with different clinicopathological features of ovarian cancer. The detail information of the correlation of CRIM1 expression with different clinicopathological features of ovarian cancer. **Supplemental Table 5.** circCRIM1 expression plasmid construction. The information of circCRIM1 expression plasmid. **Supplemental Table 6.** The sequences of shRNA targeting circCRIM1. The information of circCRIM1 shRNA. **Supplemental Table 7.** circCRIM1-FLAG expression plasmid construction. The information of circCRIM1-FLAG expression plasmid. **Supplemental Table 8.** 188aa-flag expression plasmid construction. The information of 188aa-flag expression plasmid. **Supplemental Table 9.** The detail of primer sequences. The sequences of circ primer, convergent primer1, convergent primer2 and mRNA primer. **Supplemental Figure 1.** CircCRIM1 is derived from CRIM1 by back-splicing. CircCRIM1 was generated from exon2–4 of CRIM1 (a). Sanger sequencing verified the back-splicing junction of circCRIM1 (b). CircCRIM1 was more resistant to RNase R than CRIM1 and 18 s (c). CircCRIM1 was only derived from RNA but not DNA (d).

## Data Availability

The datasets used and/or analysed during the current study are available from the corresponding author on reasonable request.

## Abbreviations

ceRNA: Competing endogenous RNA
circRNA: Circular RNA
CRIM1: Cysteine rich transmembrane BMP regulator 1
DMSO: Dimethyl sulfoxide
ORFs: Open reading frames
ZEB2: Zinc finger E-box binding homeobox 2
