# Supplementary material for: CircCRIM1 promotes ovarian cancer progression by working as ceRNAs of CRIM1 and targeting miR-383-5p/ZEB2 axis
Source: Reprod Biol Endocrinol. 2021 Nov 30;19:176. doi: 10.1186/s12958-021-00857-3 (PMC8630901; doi:10.1186/s12958-021-00857-3)
Supplement: Supplementary file 1 — Additional file 1 Supplemental Table 1. circCRIM1 expression in normal ovarian and ovarian cancer tissues. The detail information of circCRIM1 expression in normal ovarian and ovarian cancer tissues. Supplemental Table 2. CRIM1 expression in normal ovarian and ovarian cancer tissues. The detail information of CRIM1 expression in normal ovarian and ovarian cancer tissues. Supplemental Table 3. Correlation of circCRIM1 expression with different clinicopathological features of ovarian cancer. The detail information of the correlation of circCRIM1 expression with different clinicopathological features of ovarian cancer. Supplemental Table 4. Correlation of CRIM1 expression with different clinicopathological features of ovarian cancer. The detail information of the correlation of CRIM1 expression with different clinicopathological features of ovarian cancer. Supplemental Table 5. circCRIM1 expression plasmid construction. The information of circCRIM1 expression plasmid. Supplemental Table 6. The sequences of shRNA targeting circCRIM1. The information of circCRIM1 shRNA. Supplemental Table 7. circCRIM1-FLAG expression plasmid construction. The information of circCRIM1-FLAG expression plasmid. Supplemental Table 8. 188aa-flag expression plasmid construction. The information of 188aa-flag expression plasmid. Supplemental Table 9. The detail of primer sequences. The sequences of circ primer, convergent primer1, convergent primer2 and mRNA primer. Supplemental Figure 1. CircCRIM1 is derived from CRIM1 by back-splicing. CircCRIM1 was generated from exon2–4 of CRIM1 (a). Sanger sequencing verified the back-splicing junction of circCRIM1 (b). CircCRIM1 was more resistant to RNase R than CRIM1 and 18 s (c). CircCRIM1 was only derived from RNA but not DNA (d). [file 12958_2021_857_MOESM1_ESM.pdf]

Supplemental Table 1: circCRIM1 expression in normal ovarian and ovarian cancer tissues

**Groups**	**N**	**circCRIM1 expression / β-actin**	***P* value**
Normal ovarian tissues	24	0.128±0.050	***0.001***
ovarian cancer tissues	130	0.914±0.223	

Bold and Italics means P < 0.05.

Supplemental Table 2: CRIM1 expression in normal ovarian and ovarian cancer tissues

Groups	N	CRIM1 expression / β-actin	*P* value
Normal ovarian tissues	24	0.238±0.063	***0.015***
Ovarian cancer tissues	130	0.992±0.299	

Bold and Italics means P < 0.05.

Supplemental Table 3: Correlation of circCRIM1 expression with different clinicopathological features of ovarian cancer

Clinicopathological features	N	circCRIM1 expression / β-actin	*P* value
**The pathology types**			0.254
Serous carcinoma	101	0.780±0.256	
The other pathology types	29	1.382 ±0.454	
**Age**			1.000
≤ 52	67	0.914 ±0.316	
> 52	63	0.915 ±0.319	
**FIGO stages**			0.067
I-II	44	0.471 ±0.172	
III-IV	85	1.155 ±0.328	
**Pathology classification**			0.464
Well+Mod	28	0.636±0.238	
Poor	99	0.898±0.266	

Bold and Italics means $P < 0.05$, one ovarian cancer patient lacks information on stage, and three lack information on differentiation.

Supplemental Table 4: Correlation of CRIM1 expression with different clinicopathological features of ovarian cancer

Clinicopathological features	N	CRIM1 expression / β-actin	*P* value
**The pathology types**			0.338
Serous carcinoma	101	0.839 ±0.342	
The other pathology types	29	1.526 ±0.621	
**Age**			0.914
≤ 52	67	1.023 ±0.461	
> 52	63	0.959 ±0.380	
**FIGO stages**			0.110
I-II	44	0.475 ±0.202	
III-IV	85	1.261 ±0.444	
**Pathology classification**			0.073
Well+Mod	28	0.348±0.102	
Poor	99	1.023±0.359	

Bold and Italics means $P < 0.05$, one ovarian cancer patient lacks information on stage, and three lack information on differentiation.

Supplemental Table 5: circCRIM1 expression plasmid construction (The bold area are circCRIM1 sequences)

GGGAATGGAGCTATATAGCAGAGCTCGTTTAGTGACCGTCAGATCGCCTGGAGACGCCATC
CACGCTGTTTTGACCTCCATAGAAGACACCGACTCTACTAGAGGATCTATTTCCGGTGAATT
CAAAGTGCTGAGATTACAGGCGTGAGCCACCACCCCCGGCCCACTTTTTGTAAAGGTACGT
ACTAATGACTTTTTTTTTATACTTCAG**ATGAGAACTGGACTGATGACCAACTGCTTGGTT**
TTAAACCATGCAATGAAAACCTTATTGCTGGCTGCAATATAATCAATGGGAAATGTGAA
TGTAACACCATTCGAACCTGCAGCAATCCCTTTGAGTTTCCAAGTCAGGATATGTGCC
TTTCAGCTTTAAAGAGAATTGAAGAAGAGAAGCCAGATTGCTCCAAGGCCCGCTGTG
AAGTCCAGTTCTCTCCACGTTGTCCTGAAGATTCTGTTCTGATCGAGGGTTATGCTCC
TCCTGGGGAGTGCTGTCCCTTACCCAGCCGCTGCGTGTGCAACCCCGCAGGCTGTCT
GCGCAAAGTCTGCCAGCCGGGAAACCTGAACATACTAGTGTCAAAAGCCTCAGGGAA
GCCGGGAGAGTGCTGTGACCTCTATGAGTGCAAACCAGTTTTCGGCGTGGACTGCAG
GACTGTGGAATGCCCTCCTGTTCAGCAGACCGCGTGTCCCCCGGACAGCTATGAAAC
TCAAGTCAGACTAACTGCAGATGGTTGCTGTACTTTGCCAACAAGGTAAGAAGCAAGG
AAAAGAATTAGGCTCGGCACGGTAGCTCACACCTGTAATCCCAGCAGGATCCATCGATACT
AGTAAGGATCTGCGATCGCTCCGGTGCCCGTCAGTGGGCAGAGCGCACATCGCCCACAGTC
CCCGAGAAGTTGGGGGGAGGGGTCGGCAATTGAACGGGTGCCTAGAGAAGGTGGCGCGG
GGTAAACTGGGAAAGTGATGTCGTGTACTGGCTCCGCCTTTTTCCCGAGGGTGGGG

Supplemental Table 6: The sequences of shRNA targeting circCRIM1 (The bold area are the target sequences)

sh1-circCRIM1

Top strand:

GATCCG**TGCCAACAAGATGAGAACTGGAC**TTCAAGAGA**GTCCAGTTCTCATCTTGTTGGCA**TTTTTTC

Bottom strand:

AATTGAAAAAA**TGCCAACAAGATGAGAACTGGAC**TCTCTTGAA**GTCCAGTTCTCATCTTGTTGGCA**CG

sh2-circCRIM1

Top strand:

GATCCG**ACTTTGCCAACAAGATGAGAACTG**TTCAAGAGA**CAGTTCTCATCTTGTTGGCAAAGT**TTTTTTC

Bottom strand:

AATTGAAAAAA**ACTTTGCCAACAAGATGAGAACTG**TCTCTTGAA**CAGTTCTCATCTTGTTGGCAAAGT**CG

Supplemental Table 7: circCRIM1-FLAG expression plasmid construction (The bold area are 3×flag sequences)

AAAGTGCTGAGATTACAGGCGTGAGCCACCACCCCCGGCCCACTTTTTGTAAAGGTACGT
ACTAATGACTTTTTTTTTATACTTCAGATGAG**GACTACAAGGATGACGATGACAAGGAT
TACAAAGACGACGATGATAAGGACTATAAGGATGATGACGACAAA**AACTGGACTGAT
GACCAACTGCTTGGTTTTAAACCATGCAATGAAAACCTTATTGCTGGCTGCAATATAATCA
ATGGGAAATGTGAATGTAACACCATTCGAACCTGCAGCAATCCCTTTGAGTTTCCAAGTCA
GGATATGTGCCTTTCAGCTTTAAAGAGAATTGAAGAAGAGAAGCCAGATTGCTCCAAGGC
CCGCTGTGAAGTCCAGTTCTCTCCACGTTGTCCTGAAGATTCTGTTCTGATCGAGGGTTAT
GCTCCTCCTGGGGAGTGCTGTCCCTTACCCAGCCGCTGCGTGTGCAACCCCGCAGGCTGTC
TGCGCAAAGTCTGCCAGCCGGGAAACCTGAACATACTAGTGTCAAAAGCCTCAGGGAAGC
CGGGAGAGTGCTGTGACCTCTATGAGTGCAAACCAGTTTTCGGCGTGGACTGCAGGACTG
TGGAATGCCCTCCTGTTCAGCAGACCGCGTGTCCCCCGGACAGCTATGAAACTCAAGTCA
GACTAACTGCAGATGGTTGCTGTACTTTGCCAACAAGGTAAGAAGCAAGGAAAAGAATTA
GGCTCGGCACGGTAGCTCACACCTGTAATCCCAGCA

Supplemental Table 8: The introduced sequences of 188aa-flag plasmid (The bold area are 3×flag sequences)

ATGGTTGCTGTACTTTGCCAACAAGATGAGAACTGGACTGATGACCAACTGCTTGGTTTTAAACCATGCAAT
GAAAACCTTATTGCTGGCTGCAATATAATCAATGGGAAATGTGAATGTAACACCATTCGAACCTGCAGCAATC
CCTTTGAGTTTCCAAGTCAGGATATGTGCCTTTCAGCTTTAAAGAGAATTGAAGAAGAGAAGCCAGATTGCT
CCAAGGCCCGCTGTGAAGTCCAGTTCTCTCCACGTTGTCCTGAAGATTCTGTTCTGATCGAGGGTTATGCTCC
TCCTGGGGAGTGCTGTCCCTTACCCAGCCGCTGCGTGTGCAACCCCGCAGGCTGTCTGCGCAAAGTCTGCC
AGCCGGGAAACCTGAACATACTAGTGTCAAAAGCCTCAGGGAAGCCGGGAGAGTGCTGTGACCTCTATGA
GTGCAAACCAGTTTTCGGCGTGGACTGCAGGACTGTGGAATGCCCTCCTGTTCAGCAGACCGCGTGTCCCC
CGGACAGCTATGAAACTCAAGTCAGACTAACTGCAGATGGTTGCTGTACTTTGCCAACAAGA**GACTACA
AGGATGACGATGACAAGGATTACAAAGACGACGATGATAAGGACTATAAGGATGAT
GACGACAAA**TGA

Supplemental Table 9: The detail of primer sequences

Names	Sequences
circ primer	L 5'-agtgctgtgacctctatga-3'
	R 5'-ctggcttctcttcttcaattc-3'
convergent primer1	L 5'-acatctgtcgctgtaagaa-3'
	R 5'-catttcccgtccattgaga-3'
convergent primer2	L 5'-gtgctgtcccttaccca-3'
	R 5'-cagtccacgccgaaa-3'
mRNA primer	L 5'-ctctggcttatgtggtttcc-3'
	R 5'-cgcaggctggctttgt-3'

Supplemental Figure 1: CircCRIM1 is derived from CRIM1 by back-splicing

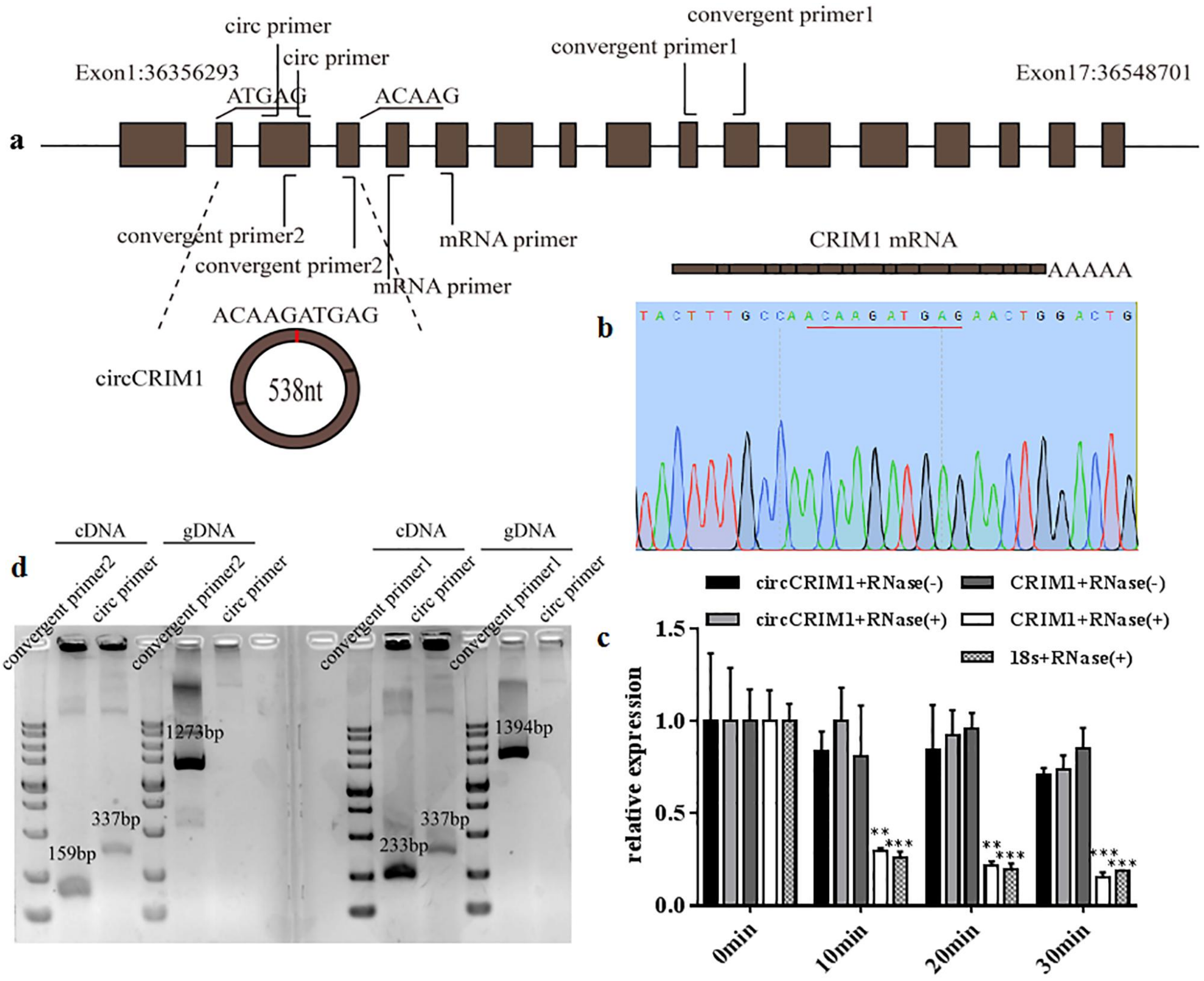

CircCRIM1 was generated from exon2-4 of CRIM1 (a). Sanger sequencing verified the back-splicing junction of circCRIM1 (b). CircCRIM1 was more resistant to RNase R than CRIM1 and 18s (c). CircCRIM1 was only derived from RNA but not DNA (d). Three separate experiments were conducted; Data are shown as the mean ± SD. *P < 0.05, **P<0.01, ***P<0.001 and ****P<0.0001.
